# Experiences of female survivors of sexual violence in eastern Democratic Republic of the Congo: a mixed-methods study

**DOI:** 10.1186/1752-1505-5-25

**Published:** 2011-11-02

**Authors:** J T Kelly, T S Betancourt, D Mukwege, R Lipton, M J VanRooyen

**Affiliations:** 1 Harvard Humanitarian Initiative, 14 Story Street, 2nd Floor, Cambridge, MA 02138, USA; 2 Harvard School of Public Health, François-Xavier Bagnoud Center for Health and Human Rights, 651 Huntington Avenue, Boston, MA 02115, USA; 3 Hôpital de Panzi, Bukavu, 8e CEPAC V/C, South Kivu, Democratic Republic of the Congo; 4 Department of Emergency Medicine, Beth Israel Deaconess Medical Center, 330 Brookline Avenue, Boston, MA 02215, USA; 5 Department of Emergency Medicine, Brigham and Women's Hospital, 75 Francis Street, Boston, MA 02115, USA

**Keywords:** conflict, Democratic Republic of the Congo, Panzi Hospital, rape, sexual violence

## Abstract

**Background:**

The conflict in eastern Democratic Republic of the Congo (DRC) is the deadliest since World War II. Over a decade of fighting amongst an array of armed groups has resulted in extensive human rights abuses, particularly the widespread use of sexual violence against women.

**Methods:**

Using a mixed-methods approach, we surveyed a non-random sample of 255 women attending a referral hospital and two local non-governmental organizations to characterize their experiences of sexual and gender-based violence (SGBV). We then conducted focus groups of 48 women survivors of SGBV to elaborate on survey findings. Quantitative and qualitative data underwent thematic and statistical analysis respectively.

**Findings:**

Of the women surveyed, 193 (75.7%) experienced rape. Twenty-nine percent of raped women were rejected by their families and 6% by their communities. Thirteen percent of women had a child from rape. Widowhood, husband abandonment, gang rape, and having a child from rape were significant risk factors for social rejection. Mixed methods findings show rape survivors were seen as "contaminated" with HIV, contributing to their isolation and over 95% could not access prophylactic care in time. Receiving support from their husbands after rape was protective against survivors' feelings of shame and social isolation.

**Interpretation:**

Rape results not only in physical and psychological trauma, but can destroy family and community structures. Women face significant obstacles in seeking services after rape. Interventions offering long-term solutions for hyper-vulnerable women are vital, but lacking; reintegration programs on SGBV for women, men, and communities are also needed.

## Background

More than a decade of fighting in eastern Democratic Republic of the Congo (DRC) has decimated the government and healthcare infrastructure in this region, creating some of the worst health and development indicators in the world. In an assessment of the health systems of 191 member countries, the World Health Organization (WHO) ranks DRC 188^th ^[1], and Oxfam and WHO estimate between 37% [2] and 75% [3] of the population have no access to healthcare.

In 1996, Laurent Kabila launched a revolution that unseated the 30-year rule of President Mobutu Sese Seko. At the same time, an influx of armed actors and refugees from the Rwandan genocide effected the complete destabilization of the country, particularly in the eastern region, where roughly 20 distinct armed groups have been identified in the past decade. A 2008 mortality study conducted by the International Rescue Committee estimates the death toll from this conflict at around 5.4 million - making it the deadliest war since World War II [4].

One of the most vicious and salient features of this conflict is the widespread sexual violence perpetrated on the women in this region. Data on the incidence of sexual violence is difficult to collect due to chronic instability, poor infrastructure, and the highly sensitive nature of rape in eastern DRC. Experts agree, however, that sexual assault is a common and pervasive form of violence in this conflict [5-8] and there are indications it has increased during the past five years [9,10]. The United Nations estimates that at least 200, 000 cases of sexual violence have been reported since the conflict started, which is thought to be a significantly low estimate [11]. In 2009, more than 15, 000 cases of sexual violence were officially reported and in 2010 there were no signs that the trend was decreasing [12]. A 2011 nation-wide survey published in the *American Journal of Public Health *found that approximately 1.69 to 1.80 million women reported having been raped in their lifetime, with women in North Kivu significantly more likely to report all types of sexual violence than those living in Kinshasa [13]. A 2010 population-based survey in eastern DRC found that 40% of women and 24% of men reported experiencing sexual violence [14].

Few studies in the peer-reviewed literature use integrated qualitative and quantitative techniques to examine sexual violence during an on-going conflict [15-17]. No studies to date have sampled from multiple service organizations and have elicited experiences and opinions about the conflict in eastern DRC from survivors of sexual and gender-based violence (SGBV) using a mixed methods approach. The objectives of this study were to characterize SGBV in eastern DRC and community reactions to it, to better understand women's experiences with SGBV services, and to gather women's suggestions for improved programming around this problem.

## Methods

We designed a mixed-methods research study, linking common themes across the survey and focus group instruments in order to facilitate comparison of qualitative and quantitative data. Women in the focus groups were also asked which issues they would like to talk about, opening up the discussion to a less structured format and giving participants an opportunity to guide the discussion.

### Quantitative Survey

#### Survey Design and Sample Selection

During July and August, 2007, women of 18 years of age or older presenting for any reason to three sites with known sexual violence programs were included in the study; respondents comprise a convenience sample of women accessing care at a large regional referral hospital in Bukavu (Panzi Hospital) and two community-based non-governmental organizations (NGOs) in the rural towns of Chambucha and Bunyakiri. The Panzi and field site respondent groups were statistically compared to determine whether significant demographic differences existed between these groups. When comparing differences between Panzi Hospital and the field sites, no significant differences were found (using t-tests and chi square tests) for age, number of children, nationality, marital status (married versus single), or education (none vs. any).

We generated a survey assessing issues related to SGBV in collaboration with local NGO staff and nurses. Professional translators translated and back translated the survey into Kiswahili; four local women then reviewed it for comprehensibility, cultural relevance, and acceptance.

The survey was designed to be appropriate for all women, whether or not they reported having experienced sexual violence. Those who answered "yes" to the question, "At any point in your life, either as a child or as an adult, have you ever been forced to have sex with someone or perform a sexual act against your will?" were coded as survivors of sexual violence and asked additional questions on their experiences.

A research team of six local female nurses was trained in research ethics and methods and carried out administration of the survey. Given low literacy rates, the surveys were administered verbally in a private setting at the clinic or NGO to ensure confidentiality.

#### Quantitative Data Analysis

Tabular data was first entered into an Excel spreadsheet and analyzed using SAS 9.2 (SAS Institute, Cary, NC). Tests of significance were calculated for crosstab comparisons (Cochrane-Mantel-Hansel chi-squares). Estimates of relative risk (odds ratios with 95% confidence intervals) were calculated for specific relationships, such as military status and rape.

### Focus Groups

#### Focus Group Design and Sample Selection

Survivors of violence of 18 years of age or older seeking medical care through the Victims of Violence Unit at Panzi Hospital during the month of January, 2008, participated in focus groups designed to explore the effects of war and violence on community and social structures, including community responses to rape, attitudes towards justice and service seeking, and proposed prevention and treatment for sexual violence. Focus group discussions were conducted in Kiswahili, the language of general communication in DRC; groups consisted of between six and 12 participants and lasted between 90 and 120 minutes.

#### Qualitative Data Analysis

A translator native to eastern DRC translated focus group audio-files and two members of the research team open-coded the transcripts [18,19]. Team members generated codes first independently then refined them collaboratively; this process allowed them to identify key unifying themes, explore complexities in the narratives, and generate hypotheses where appropriate. Codes identified as important by two or more team members defined categories; consistent variations within a category were captured as subcategories. Axial coding was used to examine relationships between categories. Data was analyzed using NVivo 8 (QSR International, Cambridge, MA).

### Human Subjects Protections

The study was approved by the institutional review board of the Harvard School of Public Health Human Subjects Committee and by all partner organizations. Verbal informed consent was obtained from research participants. Participants who had already sought services from health clinics or NGOs were chosen to ensure that they were receiving support for any physical or psychological problems resulting from rape.

### Role of the funding source

The sponsor of the study had no role in study design, data collection, data analysis, data interpretation, or writing of the report. The corresponding author had full access to all data in the study and had final responsibility for the decision to submit for publication.

## Results

All two hundred and fifty five women presenting to Panzi Hospital (n = 180) and the two rural field sites (n = 75) during the study period participated in the survey. For the qualitative study, an additional 48 women participated in five focus groups.

### Profiles of Assaults

Table 1 and Table 2 present the demographics of the study participants. No significant differences were found between hospital and field site respondents. Of the 255 women surveyed, 193 (75.7%) reported experiencing rape (Table 3); this becomes the denominator for the statistics on women's experiences with rape. These respondents were assaulted by 2.83 attackers on average (range, 1 - 10). Two-thirds of women (68.9%) reported gang rape (rape by more than one assailant on the same occasion) and 46% of women reported being abducted (forcibly taken from the place of the first rape encounter for more than one day) by their assailant. Eighty-three percent of women identified their attacker as wearing some kind of military uniform; these women were three times more likely to have been gang raped than women reporting non-uniformed attackers (OR 3.31; 95% CI 1.16--9.51; p = 0.022). Odds ratios are presented in Table 4.

**Table 1:** Demographics for Survey Respondents.*

	Number	Percentage
**Nationality:**		
Congolese	243	95.3
Other	7	2.7
No response	5	2.0
Total	255	100

**Marital Status:**		
Single	25	9.8
Married	121	47.5
Separated/divorced/abandoned	61	23.9
Widowed	45	17.6
No Response	3	1.2
Total	255	100

**Number of Children:**		
0	50	19.6
1-3	83	32.5
4-6	70	27.5
7-9	43	16.9
10+	3	1.2
Missing	6	2.4
Total	255	100

**Education Level:**		
No education	184	72.1
Primary School	54	21.2
Secondary School	16	6.3
Post - Secondary School	0	0.0
Not Specified	1	0.4
Total	255	100

**Religion**:		
Catholic	122	47.8
Protestant	110	43.1
Kimbanguist	3	1.2
Muslim	1	0.4
Other	17	6.7
No response	2	0.8
Total	255	100

**Table 2:** Age Distribution of Survey Respondents

Age range	N	Mean age	Std dev
Missing values	5	-	-

18-30	111	23.7	4.1

31-45	70	38.6	4.2

46 plus	69	53.3	6.8

Total sample Ages 18-76	255	36.1	13.2

**Table 3:** Characteristics of Sexual Assaults

	Number	Percentage
Experienced sexual assault:*		
Yes	193	75.7
No	62	24.3
No response	0	0.0
Total	255	100

**Assailant classification:**		
Stranger	169	87.6
Acquaintance	7	3.6
A friend	1	0.5
A family member	3	1.6
Husband	6	3.1
Other	1	0.5
No response	6	3.1
Total	193	100

**Military vs Civilian:**		
Civilian	23	11.9
Military	161	83.4
Other	4	2.1
No response	5	2.6
Total	193	100

**Gang:**		
Gang rape	133	68.9
Rape by 1 person	45	23.3
No response	15	7.8
Total	193	100

**Abduction:**		
Abducted	89	46.1
Not abducted	71	36.8
Other	27	14.0
No response	6	3.1
Total	193	100

**Time elapsed between Attack and Accessing Services:**		
Within 72 hours	8	4.1
Within 1 month	20	10.4
Between 2 - 11 months	75	38.8
One year or more	86	44.6
No response	4	2.1
Total	193	100

* This level of sexual violence is not an estimate of prevalence and reflects the self-selected nature of the study.

**Table 4:** Associated Odds Ratios on Characteristics of Sexual Violence

Exposure and outcome	OR	95% CIs
Of the women attacked by men in uniform (n = 151), 116 were gang raped	3.31	(1.16, 9.51)

For survivors who were widowed (n = 34), 31 reported feelings of general isolation, compared to married women	4.90	(1.27-22.10)

For women abandoned by their husbands after rape (n = 51), 44 reported feelings of general isolation	2.98	(1.10, 8.23)

Of women with children from rape (n = 98), 6 experienced community isolation	4.84	(1.41, 19.53)

Of women reporting gang rape (n = 133), 45 experienced family isolation	2.78	(1.01, 8.14)

* Associates that were tested but not found significant were: whether having a child born of rape was associated with family isolation; whether abduction was associated with family isolation; and whether gang rape was associated with community isolation.

In the focus groups, descriptions of rape were overwhelmingly mentioned in conjunction with other acts of violence related to the conflict. Combatants, and most often "foreign combatants, " such as soldiers from Rwanda, were blamed for the attacks on women. Gang rape and rape in public were often described as occurring in tandem with village attacks by armed men that also involved looting, rape with instruments such as guns, abduction of the victim, forced incest (an assailant forcing a family member such as a father or brother to have sex with the victim), and injury or execution of family members.

### Rejection

As a result of rape, 29% of women were rejected by their families and 6.2% by their communities (Table 5). Family rejection means that a woman is told she can no longer stay in the home of her husband or parents. In the case of community rejection, women are ostracized by peers to such a degree they feel forced to leave the community.

**Table 5:** Rape Survivors' Experiences with Rejection

	Number	Percentage
**Experienced rejection from family**		
Yes	55	28.5
No	116	60.1
No response	22	11.4
Total	193	100

**Experienced rejection from community**		
Yes	12	6.20
No	116	60.1
No response	65	33.7
Total	193	100

Of the 193 women who reported rape, those who were widowed were almost five times as likely to report feelings of general isolation compared to married women (OR 4.90, 95% CI 1.27 - 22.10; p = 0.016). Women abandoned by their husbands were almost three times as likely to report feelings of general isolation compared to non-abandoned married women (OR 2.98, 95% CI 1.02 - 8.32; p = 0.029). Gang-raped women wereroughly three times more likely to experience rejection from their familycompared to women who were not gang-raped (OR 2.78, 95% CI 1.01--8.14; p =0.049).

In all focus groups, the issue of stigmatization and rejection of survivors of violence arose spontaneously and remained a dominant theme throughout the discussions. Stigmatization in the community was often expressed as gossip or "finger pointing" (*kushota kidole *in Kiswahili), which intensified survivors' feelings of shame and humiliation. Women described how often local mores created an environment conducive to the stigmatization of survivors: customs that had previously been directed towards female adulterers were now applied to victims of rape. Women who have sex outside of marriage, whether voluntarily or by force, were perceived to bring misfortune to the household.

Women in each focus group talked specifically about being rejected by their husbands; one of the most commonly cited reasons for this was a husband's fear of disease and "contamination" from his wife. Describing men's reactions, one woman said, "They repudiate us. They know that we have been raped and that we have been infected. So to save their lives they abandon us."

Women in eastern DRC also suffer from traumatic fistulas resulting from violent rape and obstetric fistulas resulting from lack of prenatal care. Women described fistulas as another risk factor for rejection:

"Your husband will not accept to stay with such wife whereas there [are] many other women who are in a good health.... He will tell you that he can not live with a wife who is wetting his bed every day."

In some focus groups, gang rape was specifically mentioned in relation to family rejection, confirming the survey finding. One woman summarized the phenomenon: "Your husband will say he cannot keep a woman who has been raped by the whole battalion, and he will repudiate you. When you go to your parents' house, they will ask you why you have destroyed your marriage."

Women offered an explanation as to why rejection by one's husband increased the likelihood of feelings of general isolation. As one women said, "Your husband is the first person to reject you [after rape], and then comes your family." Women noted that in cases when a man truly loves his wife, however, he may choose to assist rather than repudiate her. Women said this kind of support can act as a powerful resource to help the survivor resist community stigmatization: "[Men] can help survivors get respect from other people, because if your husband doesn't humiliate you, other people won't."

Even if they are not rejected, however, participants described cycles of blame and anger that can develop between a husband and wife after rape. Women may face increased risk of abuse or neglect within the home after rape. As one woman said, "Even if your husband is not looking for other women [after the rape], he won't take care of you any more; even your own children can start thinking that you have been contaminated."

### Children from Rape

Thirteen percent of women had a child from rape. Women who did have children from rape were almost five times as likely to report experiencing community isolation than those who did not report having children from rape (OR 4.84; 95% CI 1.41 - 19.53; p = 0.021).

In focus groups, women stated that having a child from rape was cited as one of the reasons a woman might be isolated by her family or community. In the majority of groups, women specifically mentioned that when one has a child from rape, the father is "unknown" - a phenomenon that leaves the women alone to shoulder the responsibility of raising the child and face negative community reactions.

### Services

Almost half of women polled (44.6%) waited a year or more before seeking SGBV services. Fifty-five percent of women stated it took them more than a day to travel to SGBV service locations; only 4.2% of the women received SGBV services within 72 hours of the attack - a medically important window of time in which victims can be given prophylaxis for sexually transmitted infections (STIs) and HIV.

Discussing the services that would be most helpful, women were extremely positive about educational programs to help communities understand how to accept survivors of rape. Additionally, a form of marriage counseling was suggested to help husbands and wives move past the trauma of the attack. As one woman said, "There is a need to teach both husband and wives, to tell them that what occurred was just an accident that neither of them wanted to happen."

Women also noted that one way to help them reintegrate back into their families, or to prevent their husbands from rejecting them at all, was to prove they were not infected with HIV or STIs. For this reason, STI testing was described as an extremely important intervention that may act as a first step towards reintegration. One woman explained: "[Service organizations] can show [your husband] documents you got from the hospital proving that you are not infected. In that situation he can accept to take you back home. But if you are infected he will repudiate you immediately."

### Justice

When asked whether they would like to see their attacker arrested for the crime of rape, 58% of women said they would. Women who answered "no" often expressed skepticism about the capacity of the police force to carry out arrests rather than a lack of desire to see their attacker punished. A smaller proportion of women (48.2%) responded "yes" to the question: "Would you personally be willing to pursue legal action against your assailant?"

Focus group discussions strongly emphasized that perpetrators of rape deserve to be punished. Participants stressed, however, that the Congolese government was ineffective and the judiciary system was flawed and corrupt. As one woman said, "We are slaves of [armed groups]. They can do whatever they like and we can't do anything about it."

Women also spoke about the risk of "revenge attacks, " including rape or murder, once a perpetrator is released from prison. These may be perpetrated by the accused, but also by men who do not want women speaking out against sexual violence:

"Q: Is it possible that a woman who has been raped will be raped again when she gets back to her village?

R: Of course! People would say this woman who went back is the one who gave the information about [her assailants], and then they will come and kill you."

## Limitations

Survivors of sexual violence are a difficult population to reach, and conducting research on this group is ethically complex. This reality poses a number of study limitations. Women surveyed at service points may represent those with the most serious medical issues and disabilities arising from rape. It is possible, however, that women who have not come forward to seek services are among the most vulnerable and disadvantaged. They may be unable to access care because of insecurity or the inability to travel due to distance or poverty. This makes it difficult to determine the direction of possible bias in the estimates of rejection, stigmatization, and types of violence. This study, drawn from a non-random sample, cannot therefore be used to generalize about women's experiences with sexual violence in eastern DRC or determine the prevalence of rape in the general population.

Given that a significant number of respondents waited more than a year to seek services for rape, recall bias may have influenced focus group results. Research participants were informed that their responses would not directly affect services they might receive currently or in the future in order to further reduce bias in the responses. Facilitators were trained in focus group methods before the study and through on-going debriefs during the research period in an attempt to mitigate undue influence by moderators on participant responses. In qualitative research, the investigator will inevitably have a role in influencing the interpretation and analysis of the data. During the qualitative analysis phase, the research team maintained an on-going dialogue and continued to re-examine hypotheses and refine themes to address the possible biases inherent in having only one person interpret the data.

## Discussion

These results emphasize the brutality of the sexual violence being carried out in eastern DRC. Levels of gang rape and abduction seem to be higher in eastern DRC than in other recent conflicts that were also characterized by high rates of sexual violence. Prolonged violence, impunity, and a proliferation of armed groups may all contribute to the extremely widespread and violent forms of rape seen in DRC. In both survey and focus group results, women noted that gang rape and abduction are salient features of the sexual violence in eastern DRC, and that the majority of attacks are carried out by men in uniform. These results are in line with other estimates from the region. A 2003 report by Réseau des Femmes pour un Dévelopement Associatif (RFDA) found that 79% of women reported gang rape and 26% reported rejection by their family [20]. Data gathered by Médecins Sans Frontières in the Baraka province, from 2000-2004, found that 75% of women reported gang rape [21]. Good epidemiologic and demographic information on types of violence perpetrated in other recent conflicts is lacking. However, gang rape and abduction in the DRC seems to be more common than in many other recent conflicts such as Sierra Leone, where a population-based study from Physicians for Human Rights found 33% of survivors had been gang raped and 33% abducted [22].

Survivors of sexual violence are viewed with intense negativity, putting them at significant risk of being rejected from their own family or community. Gang rape, and the associated perception that women are "contaminated, " increased women's risk of being abandoned by their husbands. To address this, women suggested marriage counseling to help both husband and wife move past the trauma of the attack, perhaps occurring in tandem with presenting results from a woman's STI and HIV tests to her husband. Only women who test negative for STIs and HIV, however, were seen by participants as likely candidates for reintegration, begging the question of how to best provide services for women who test positive. One in 10 women had a child from rape, which significantly increased her risk of social isolation. These women, like women who test positive for STIs and HIV and women who have fistula, are highly vulnerable populations that need specialized, long-term services that are currently lacking in this context. Results of a 2011 qualitative study with community leaders in eastern DRC [23] reinforced certain findings from this study, including the fact that survivors of violence are perceived as being HIV positive after rape, contributing to their isolation. Integrating religious and community leaders into programs that respond to negative attitudes towards survivors will be vital in addressing the stigma towards these women.

Survey and focus groups results highlighted how marriage protects against feelings of isolation. More work is needed to understand the risk factors that make a husband likely to abandon his wife after rape; to identify possible intervention points; and to elaborate the effects of rape on a woman's livelihood and future. Focus group results illustrate that women who are not rejected outright may still face significant problems after the rape, and that counseling for both husband and wife is needed. More work needs to be done to understand how economic interventions, for both women and men, can facilitate social reintegration for vulnerable populations and facilitate community-level post-conflict recovery.

By employing mixed quantitative and qualitative methods, this study attempts to triangulate the most salient research findings across different modes of inquiry and to explore the phenomenon and its consequences. The focus group narrative data contextualizes and supports the quantitative analysis using women's own experiences and opinions. Qualitative data also help reveal important areas for future inquiry such as improved reintegration programming incorporating members of a woman's family and the need to better understand the role of men in experiencing, preventing, and perpetrating violence.

Sexual violence in eastern DRC is a complex and highly destructive feature of the conflict that creates profound physical, emotional, and social distress. Services to address the manifold social and medical consequences of rape are inadequate, and stigma and fear of reprisal attacks may keep women from seeking out the health and legal services that are available. More mixed-methods research in conflict and post-conflict environments could help illuminate complex phenomena like sexual violence using qualitative data from affected populations to inform quantitative methods.

## Competing interests

The authors declare that they have no competing interests.

## Authors' contributions

JK designed the study and data collection tools; oversaw the collection of the survey and focus group data; and wrote the first draft of the paper. TB reviewed the qualitative data analysis write-up. DM helped design the quantitative and qualitative survey instruments and aided with the administration of the study at Panzi Hospital. RL conducted the quantitative data analysis and assisted in writing the quantitative analysis section of the paper. MVR provided guidance in the analysis and interpretation of results and in the writing of the paper. All authors criticised drafts of the paper, and JK was responsible for subsequent collation of inputs and redrafting. All authors read and approved the final manuscript.
